# Expression of concern: Design and development of highly sensitive PEDOT-PSS/AuNP hybrid nanocomposite-based sensor towards room temperature detection of greenhouse methane gas at ppb level

**DOI:** 10.1039/d3ra90018e

**Published:** 2023-03-16

**Authors:** Syed Khasim, Apsar Pasha, Nacer Badi, Adnen Ltaief, S. A. Al-Ghamdi, Chellasamy Panneerselvam

**Affiliations:** a Department of Physics, Faculty of Science, University of Tabuk Tabuk-71491 Kingdom of Saudi Arabia syed.pes@gmail.com; b Renewable Energy Laboratory, Nanotechnology Research Unit, University of Tabuk Tabuk-71491 Kingdom of Saudi Arabia; c Department of Physics, Ghousia College of Engineering Ramanagaram-562159 Karnataka India; d Department of Biology, Faculty of Science, University of Tabuk Tabuk-71491 Kingdom of Saudi Arabia

## Abstract

Expression of concern for ‘Design and development of highly sensitive PEDOT-PSS/AuNP hybrid nanocomposite-based sensor towards room temperature detection of greenhouse methane gas at ppb level’ by Syed Khasim *et al.,* RSC *Adv.*, 2021, **11**, 15017–15029. DOI https://doi.org/10.1039/D1RA00994J.

*RSC Advances* is publishing this expression of concern in order to alert our readers that we are presently unsure of the reliability of the data reported in Fig. 3 of the article. Fig. 3b of this article is identical to Fig. 3a of ref. 1.

An investigation is underway, and an Expression of Concern will continue to be associated with the article until a final outcome is reached.

Laura Fisher

9th March 2023

Executive Editor, *RSC Advances*

## Supplementary Material
